# Cluster-Based Maximum Consensus Time Synchronization for Industrial Wireless Sensor Networks †

**DOI:** 10.3390/s17010141

**Published:** 2017-01-13

**Authors:** Zhaowei Wang, Peng Zeng, Mingtuo Zhou, Dong Li, Jintao Wang

**Affiliations:** 1 Key Laboratory of Networked Control System, Shenyang Institute of Automation, Chinese Academy of Sciences, Shenyang 110016, China; wangzhaowei@sia.cn (Z.W.); lidong@sia.cn (D.L.); wangjintao@sia.cn (J.W.); 2 University of Chinese Academy of Sciences, Beijing 100049, China; 3 Key Laboratory of Wireless Sensor Network & Communication, Shanghai Institute of Microsystem Information and Technology, Chinese Academy of Sciences, Shanghai 200050, China; zhoumingtuo@hotmail.com; 4 Shanghai Research Center for Wireless Communications, Shanghai 201210, China

**Keywords:** time synchronization, convergence rate, industrial wireless sensor networks, maximum consensus, communication delays

## Abstract

Time synchronization is one of the key technologies in Industrial Wireless Sensor Networks (IWSNs), and clustering is widely used in WSNs for data fusion and information collection to reduce redundant data and communication overhead. Considering IWSNs’ demand for low energy consumption, fast convergence, and robustness, this paper presents a novel Cluster-based Maximum consensus Time Synchronization (CMTS) method. It consists of two parts: intra-cluster time synchronization and inter-cluster time synchronization. Based on the theory of distributed consensus, the proposed method utilizes the maximum consensus approach to realize the intra-cluster time synchronization, and adjacent clusters exchange the time messages via overlapping nodes to synchronize with each other. A Revised-CMTS is further proposed to counteract the impact of bounded communication delays between two connected nodes, because the traditional stochastic models of the communication delays would distort in a dynamic environment. The simulation results show that our method reduces the communication overhead and improves the convergence rate in comparison to existing works, as well as adapting to the uncertain bounded communication delays.

## 1. Introduction

Industrial Wireless Sensor Networks (IWSNs) utilize wireless communication to transmit data between industrial measurement and control equipment, which can effectively increase the coverage area of the network, reduce the layout cost, and improve the speed of the network construction [1,2,3]. In IWSNs, many applications rely heavily on a common notion of time, such as nodes sleep scheduling, transmission scheduling, Time Division Multiple Access (TDMA)-based communication, sensed data fusion, etc. However, nodes’ times are usually different from each other, and the reason is as follows: (1) each node’s time is obtained by counting the output pulse of an internal crystal oscillator, which is seriously affected by the crystal oscillator’s accuracy; (2) the clock skew—namely, the timing rate of each node’s clock—is different, because of the difference of intrinsic properties between crystal oscillators [4,5] and factory environment interference (such as temperature and humidity variation, and electromagnetic interference); (3) initial clock offset—namely, the difference of each node’s online time. Therefore, time synchronization algorithm is necessary in IWSNs.

Some special applications (such as fault tracking of the equipment and co-operation of industrial robots) have put forward special requirements for time synchronization in IWSNs: (1) low-power, because most of the nodes in IWSNs are battery powered, which is similar to wireless sensor networks; (2) high-precision, which is the primary demand to meet the high real-time in industrial production control process; (3) fast convergence: it should meet the requirements of rapid detection and control when equipment is on-line, and fast-recovery after node failure [6].

Currently, most of the time synchronization methods in wireless sensor networks mainly aim to improve the precision and reduce the energy consumption [6,7,8,9,10,11], but they seldom consider the synchronization convergence time [12] and take the influence of network structure into account, which is important for industrial applications. In addition, information exchange is the key basis of time synchronization methods. In real environments, the communication delays between nodes cannot be ignored. Many methods suppose that the communication delays among two nodes are symmetric or obey a particular distribution, such as exponential distribution, Gaussian distribution, and so on [12,13,14,15]. They also neglect the fact that the predefined delay model would change with the influence of external environment, which results in a negative effect on synchronization performance, such as accuracy and convergence time of sync methods. Clustering technology has been widely used to design time synchronization methods [16,17,18]. Normally, the computation, storage, communication, and power supply ability of a cluster-head is larger than ordinary nodes. So, a cluster-head can bear more calculation and communication in sync methods to prolong the network lifetime.

In view of the above problems, we develop a sync protocol for cluster-based IWSNs. The key idea of our method is to employ a maximum consistency to perform time synchronization instead of average consistency, which needs more iterations. The synchronization process is activated by the cluster head instead of all the nodes in order to save the energy of ordinary nodes. Then IWSNs diffuse the synchronous state with the assistance of overlay nodes, and realize time synchronization cluster by cluster, rather than point to point. Furthermore, two non-decreasing and non-increasing functions are introduced to make the convergence of logic skew and offset come true under communication delays, respectively.

The contributions of our work are summarized as follows:

(1) We first ignore the communication delays and propose a novel Cluster-based Maximum consensus Time Synchronization (CMTS) method in combination with the characteristics of max-consistency theory and overlapping cluster network structure.
(2) Different from the traditional communication delay models, the bounded delay model has been proposed in [19,20], which is more realistic and reasonable. Then, a Revised-CMTS is further given by combining with the current works against the bounded communication delays.
(3) The performance analysis and simulation results demonstrate that our protocol could effectively reduce the communication overhead, speed up the convergence time, and improve the synchronization precision. Additionally, CMTS needs at most three times send-receive process to achieve intra-cluster synchronization, with linear time to achieve inter-cluster synchronization. The convergence time of Revised-CMTS heavily depends on the probability that the lower bound and upper bound of communication delays appear successively; namely, a higher probability generates a faster convergence speed.

The rest of this paper is organized as follows. Section 2 describes the work related to time synchronization in IWSNs, followed by the clock model in Section 3. In Section 4, we propose and analyze the CMTS method and Revised-CMTS method. The performance analysis and simulation results are shown in Section 5. Finally, we present the conclusion and future work in Section 6.

## 2. Related Work

At present, a variety of time synchronization methods have been proposed on the basis of different network structures: traditional network structure and clustering network structure.

In traditional network structures (including mesh, star and hierarchical structures), representative sync methods include Reference Broadcast Synchronization (RBS) [7], Timing-sync Protocol for Sensor Networks (TPSN) [8], Flooding Time Synchronization Protocol (FTSP) [9], etc. In the above methods, ordinary nodes synchronize quickly with the source node by exchanging time information. However, the existence of a time source would induce a single point failure problem. Furthermore, the synchronization error seriously accumulates with the increasing of hop count, which would bring poor scalability. So, some new methods are also designed. For instance, Yildirim et al. [10] proposed a Flooding with Clock Speed Agreement (FCSA) protocol which is designed to provide skew synchronization between neighbors, and this protocol can reduce the synchronization error that accumulates with the increasing of hop in FTSP. Cho et al. [11] developed a method to maintain accurate time by adopting hardware-assisted time stamp and drift correction. Besides, for consensus-based methods Average TimeSync (ATS) [21] and Maximum Time Synchronization (MTS) [12], there is no time source. Local nodes utilize their neighbor’s time information to achieve logic clock sync. MTS protocol is built on an asynchronous consensus theory. The main idea is to maximize the local information to achieve a global synchronization, and, ATS protocol is based on a cascade of two average consensus algorithms for skew and offset synchronization. Unlike ATS, the distributed clock synchronization scheme [13] is based on the two-way message exchange model and group averaging instead of point-to-point averaging. So, the robustness and scalability of consensus-based methods are superior to the time source-based ones, but with more energy consumption.

Clustering topology makes the communication hop manageable and eliminates the redundant communication compared with the traditional structures, especially in IWSNs. Therefore, incorporating clustering technique into synchronization method is a more effective way to reduce the energy consumption and improve the convergence speed of the sync algorithm. For instance, Yadav et al. [16] implemented Cluster Based Hierarchical-Flooding Time Synchronization algorithm (CBH-FTS), which is driven by semantic. In CBH-FTS, TPSN and FTSP are modified to fulfill the needs of time synchronization in cluster-based hierarchical WSNs. Cluster-based Time Synchronization for WSN (CSSN) [17]—which is similar to CBH-FTS, includes two phases—intra-cluster synchronization phase and inter-cluster synchronization phase. Because of the existence of time source and hierarchical structure, there is no doubt that CBH-FTS and CSSN would inherit the shortcomings, and additional communication overhead is needed to maintain the network topology.

With the idea of clustering and consistency theory, Wu et al. [18] proposed a Clustered Consensus Time Synchronization (CCTS) algorithm, which is most relevant to our work. Based on a linear model of average consensus algorithm, CCTS is also divided into two phases to achieve global synchronization—namely, intra-cluster and inter-cluster synchronization. However, CCTS’s inter-cluster synchronization is started after achieving intra-cluster synchronization, and the offset compensation is started after applying the skew compensation either in intra-cluster synchronization or inter-cluster synchronization, which results in a lower convergence rate. Meanwhile, the convergence rate of CCTS is also closely related to the initial synchronization error, which causes more iterations.

However, all of the above works seldom consider the communication delays that occur when a packet is sent after time-stamping and received at the moment in which the packet is time-stamped, which has a great impact on the accuracy and convergence time of sync algorithms. Although many current works have been done to deal with the time delay of the Markovian jump linear systems [22,23,24,25], they cannot be directly applied to time synchronization methods. So, many others methods have been presented against the communication delays. Wang et al. [14] utilized the overhearing mechanism to realize synchronization with time source, but it regards the link delay as Gaussian distribution. Sun et al. [15] supposed the link delay obeys exponential distribution, and abstracted the problem of clock parameters estimation into a minmax-optimization issue, and a minimum variance unbiased estimator is developed to solve the problem. However, the uncertain communication delays vary with the change of the external factors. A single predefined model could not accurately describe the delay. Consequently, Garone et al. [19,20] proposed a Robust Average TimeSync (RoATS) to improve the performance of traditional ATS method [21]. The papers assumed that there exists a lower bound on the minimum interval between the transmission of two consecutive packets and an upper bound on the maximum delay for the reception of a packet. At the same time, for a couple of nodes, they update their clock parameters with same value, but in opposite direction. He et al. [26] developed a distributed time synchronization protocol by adapting the concept of maximum consensus under bounded noise in WSNs, which is similar to RoATS, but with a higher accuracy and faster convergence speed. However, RoATS is the expansion of ATS, which cannot simultaneously synchronize the skew and offset. For [26], each node needs to take a lot of computation and storage, which results in more energy consumption.

Therefore, we first proposed CMTS without considering the communication delays. Then, a Revised-CMTS was developed by reference to the current work [26], with reliable guarantee on the synchronization accuracy and convergence against bounded communication delays. Compared with the aforementioned methods, our method reduces the transmission of ordinary nodes and balances energy consumption of nodes in the network.

## 3. Clock Model

First, let us define the clock model involved in the problem before introducing the proposed method. In general, each node’s time $\tau \left(t\right)$ is obtained by counting the output pulse of the internal crystal oscillator, which is different from the absolute time *t* because of the existence of skew deviation and offset error. By reference to [12,18], $\tau \left(t\right)$ of node *i* is an increasing function of *t*, which is given by

(1) $$\tau_{i}\left(t\right)=\alpha_{i}t+\beta_{i},$$

where $\alpha_{i}$ and $\beta_{i}$ are the local clock skew and offset, which determine the clock speed and the initial synchronization error, respectively. However, $\alpha_{i}$ and $\beta_{i}$ are unknown to the node and cannot be adjusted directly, because the absolute time *t* is unavailable to the node. So, we introduce the skew and offset compensation parameters to adjust the local time and build the logic clock, which is given by

(2) $${\hat{\tau }}_{i}\left(t\right)={\hat{\alpha }}_{i}\left(t\right)\tau_{i}\left(t\right)+{\hat{\beta }}_{i}\left(t\right)={\hat{\alpha }}_{i}\left(t\right)\alpha_{i}t+\left({\hat{\alpha }}_{i}\left(t\right)\beta_{i}+{\hat{\beta }}_{i}\left(t\right)\right),$$

where ${\hat{\alpha }}_{i}$ and ${\hat{\beta }}_{i}$ are the skew and offset compensation parameters of node *i* respectively, which can be updated via time synchronization algorithm.

In this paper, consensus time synchronization’s purpose is to update ${\hat{\alpha }}_{i}$ and ${\hat{\beta }}_{i}$ to achieve logic time synchronization; namely,

(3) $$\left\{\begin{array}{c} \;\lim_{t\to \infty }{\hat{\alpha }}_{i}\left(t\right)\alpha_{i}=\alpha_{\nu },\;\;\;\\ \lim_{t\to \infty }{\hat{\alpha }}_{i}\left(t\right)\beta_{i}+{\hat{\beta }}_{i}\left(t\right)=\beta_{\nu }, \end{array}\right.$$

where $\alpha_{\nu }$ and $\beta_{\nu }$ are the skew and offset of the global logic clock, so we can obtain the global logical clock $\tau_{\nu }\left(t\right)=\alpha_{\nu }t+\beta_{\nu }$. Here, we should notice that the global logical clock is just a common logic reference clock, rather than an external clock source. Clock parameters ($\alpha_{\nu }$, $\beta_{\nu }$) can be the average value or extreme value of the nodes’ clock, which has a huge impact on the synchronization performance of the time synchronization algorithm. As we all know, the extreme value of clock parameters can be easily acquired via only one comparison, but the acquisition of an average value needs multiple iterations. In addition, the extreme value is a fixed value, while the average value is just an approximate value. So, we adopt a maximum value-based consensus time synchronization algorithm in this paper, which results in a faster convergence rate and higher synchronization accuracy than average value-based algorithms.

## 4. CMTS and Revised-CMTS Methods

IWSN is modelled as a graph G=(V,E), where V={1,2,⋯,n} is the set of nodes, and (i,j)∈E indicates that node *i* and node *j* can communicate with each other. In clustered IWSNs, $G_{C}=\left(V_{C},E_{C}\right)$, where $V_{C}=\left\{C_{i}|i=1,2,\cdots ,m\right\}$ is the set of clusters, and $\left(C_{i},Cj\right)\in E_{C}$ indicates that cluster $C_{i}$ and $C_{j}$ can exchange messages via overlap nodes. Therefore, $V={⋃}_{i\in (1,m)}C_{i}$. Table 1 gives some important notations.

**Figure 1:**
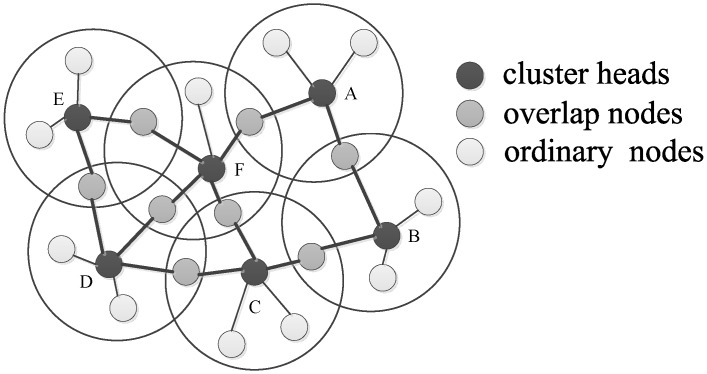
Overlapping cluster-based topology of Industrial Wireless Sensor Networks (IWSNs).

As shown in Figure 1, a cluster includes cluster head and cluster member nodes, including overlap nodes and non-overlapped ordinary nodes. Cluster-heads communicate with each other via the overlap nodes, and ordinary cluster member nodes just communicate with their cluster heads in single hop. In the time synchronization of overlapping cluster-based IWSNs, a cluster head transmits its time information to its member by radio, and cluster members do not need to transmit time information to all of the neighbors, which can improve the transmission efficiency and channel utilization, and further save the communication overhead and reduce the energy consumption.

### 4.1. CMTS Method

As shown in Algorithm 1, we develop the CMTS algorithm based on the IWSNs structure, communication mechanisms, and the pre-described clock model. Different from the traditional distributed time synchronization algorithms, the CMTS algorithm is started by the cluster heads rather than all of the nodes. Based on the hardware clock reading, cluster heads start the synchronization process in IWSNs periodically with a preset sync interval. The cluster head *h* broadcasts its time information, which includes hardware clock value $\tau_{h}\left(t\right)$, skew compensation parameter ${\hat{\alpha }}_{h}\left(t\right)$, and offset compensation parameter ${\hat{\beta }}_{h}\left(t\right)$ to its cluster members. When the cluster member *i* receives its cluster head *h*’s information, it will record the current reading of its hardware clock $\tau_{i}\left(t\right)$, clock compensation parameters ${\hat{\alpha }}_{i}\left(t\right)$ and ${\hat{\beta }}_{i}\left(t\right)$, and send back the information to its cluster head *h*. Now, one time information exchange is completed.

Local node *l* will update its clock compensation parameters based on the latest time information received from node *j*, when it has a historical record from the same node. Meanwhile, node *j* is the cluster member when node *l* is the cluster head. Otherwise, node *j* is the cluster head when node *l* is the cluster member. Here, the update rules are designed to obtain the maximum value of ${\hat{\alpha }}_{l}\left(t\right)\alpha_{l}$ and ${\hat{\alpha }}_{j}\left(t\right)\alpha_{j}$ firstly [12,27]; namely,

(4) $${\hat{\alpha }}_{l}\left(t^{+}\right)\alpha_{l}\leftarrow max\left({\hat{\alpha }}_{l}\left(t\right)\alpha_{l},{\hat{\alpha }}_{j}\left(t\right)\alpha_{j}\right)$$

while, the result is seriously dependent on the relative clock skew $\alpha_{j}/\alpha_{l}$, i.e., ${\hat{\alpha }}_{l}\left(t^{+}\right)\leftarrow max\left({\hat{\alpha }}_{l}\left(t\right),{\hat{\alpha }}_{j}\left(t\right)\alpha_{j}/\alpha_{l}\right)$. It should be noted that Media Access Control (MAC) layer time stamping is used to eliminate the uncertain delay in sensor networks [28], and we suppose the communication delays are constant. So, in CMTS algorithm, the relative skew $\alpha_{j}/\alpha_{l}=\Delta S_{j}/\Delta S_{l}=\left(\tau_{j}\left(t_{k}\right)-\tau_{j}\left(t_{k-1}\right)\right)/\left(\tau_{l}\left(t_{k}\right)-\tau_{l}\left(t_{k-1}\right)\right)$, and the logic offset compensation parameter will be updated according to Algorithm 1, with the purpose of achieving the maximum value of logic offset.

**Algorithm 1** CMTS algorithm(1). In clustered IWSNs, set ${\hat{\alpha }}_{i}=1$ and ${\hat{\beta }}_{i}=0$ for each node, and set the sync interval *T* for cluster heads. (2). For cluster head *h*, if $\tau_{h}\left(t\right)=kT$, $k\in N^{+}$, node *h* broadcasts < $\tau_{h}\left(t_{k}\right)$, ${\hat{\alpha }}_{h}\left(t_{k}\right)$, ${\hat{\beta }}_{h}\left(t_{k}\right)$ > to its cluster members. Upon receiving the time information from cluster head, cluster member *i* records current information < $\tau_{i}\left(t_{k}\right)$, ${\hat{\alpha }}_{i}\left(t_{k}\right)$, ${\hat{\beta }}_{i}\left(t_{k}\right)$ >, and sends it back to cluster head *h*. (3). When node *l*—which can be the cluster head or cluster member—receives time information from cluster member or cluster head *j*, and has a historical record < $\tau_{j}\left(t_{k-1}\right)$, ${\hat{\alpha }}_{j}\left(t_{k-1}\right)$, ${\hat{\beta }}_{j}\left(t_{k-1}\right)$ >, then compute
$$\Delta S_{j}\leftarrow \tau_{j}\left(t_{k}\right)-\tau_{j}\left(t_{k-1}\right).$$
$$\Delta S_{l}\leftarrow \tau_{l}\left(t_{k}\right)-\tau_{l}\left(t_{k-1}\right).$$ Caes1: if ${\hat{\alpha }}_{j}\Delta S_{j}≻{\hat{\alpha }}_{l}\Delta S_{l}$, then
$${\hat{\alpha }}_{l}\left(t_{k}^{+}\right)\leftarrow \frac{{\hat{\alpha }}_{j}\left(t_{k}\right)\Delta S_{j}}{\Delta S_{l}}.$$
$${\hat{\beta }}_{l}\left(t_{k}^{+}\right)\leftarrow {\hat{\alpha }}_{j}\left(t_{k}\right)\tau_{j}\left(t_{k}\right)+{\hat{\beta }}_{j}\left(t_{k}\right)-{\hat{\alpha }}_{l}\left(t_{k}^{+}\right)\tau_{l}\left(t_{k}\right).$$ Case2: if ${\hat{\alpha }}_{j}\Delta S_{j}={\hat{\alpha }}_{l}\Delta S_{l}$, then
$${\hat{\beta }}_{l}\left(t_{k}^{+}\right)\leftarrow max_{i=l,j}\left({\hat{\alpha }}_{i}\left(t_{k}\right)\tau_{i}\left(t_{k}\right)+{\hat{\beta }}_{i}\left(t_{k}\right)\right)-{\hat{\alpha }}_{l}\left(t_{k}^{+}\right)\tau_{l}\left(t_{k}\right).$$ Case3: if ${\hat{\alpha }}_{j}\Delta S_{j}<{\hat{\alpha }}_{l}\Delta S_{l}$, then continue with step 4. (4). Node *l* store the latest time information($\tau_{l}\left(t_{k}\right)$, $\tau_{j}\left(t_{k}\right)$).

In intra-cluster time synchronization, it is obvious that we can achieve synchronization by using CMTS method at most three times message exchange, namely in a finite time slot $T_{S}$. So, we can obtain the Theorem 1 to realize all nodes’ sync.

**Theorem** **1.** *The global logic skew and offset will converge by using CMTS method in inter-cluster time synchronization; namely,*

(5) $$\left\{\begin{array}{l} \alpha_{\nu }={max}_{i\in V}\alpha_{i},\;\;\\ \beta_{\nu }={max}_{i\in V_{max}}\beta_{i}, \end{array}\right.$$

*where $V_{max}$ denotes the set of nodes whose global logic skew has achieved $\alpha_{max}$ (*i.e.,*$\tau_{\nu }\left(t\right)=\alpha_{\nu }t+\beta_{\nu }$), and the convergence time satisfies:*

(6) $$T_{\mathrm{cov}}^{CMTS}\leq mT_{S}.$$

**Proof** **of Theorem 1.** Here, $N_{\nu }\left(t\right)$ denotes the number of nodes belonging to the set $V_{\nu }\left(t\right)$ at time *t*, and $V_{\nu }\left(t\right)$ denotes the set of nodes whose logical clock equals to $\tau_{\nu }\left(t\right)$. At the initial stage, there is at least one node whose global logic clock skew and offset equal to $\alpha_{\nu }$ and $\beta_{\nu }$ obviously; i.e., $N_{\nu }\left(0\right)\geq 1$, $V_{\nu }\left(0\right)\neq \emptyset$. So, at the time interval $\left(0,T_{S}\right)$, at least one cluster $C_{i}$ could achieve intra-cluster time synchronization; i.e., $V_{\nu }\left(T_{S}\right)=C_{i}$, $N_{\nu }\left(t\right)\geq \left|C_{i}\right|$, where $\left|\cdot \right|$ is the cardinality of set $C_{i}$ . Similarly, at the time interval $\left(\left.T_{S},2T_{S}\right]\right.$, there exists at least a neighbor cluster $C_{j}$ who can synchronize with cluster $C_{i}$ via overlap nodes $v\in C_{i}\cap C_{j}$, i.e., $V_{\nu }\left(2T_{S}\right)=C_{i}\cup C_{j}$, $N_{\nu }\left(2T_{S}\right)\geq \left|C_{i}\cup C_{j}\right|$. This means that $N_{\nu }\left(t\right)$ is non-decreasing, and so on, when $t\geq mT_{S}$, $V_{\nu }\left(t\right)=\cup_{i\in \left(1,m\right)}C_{i}$; hence, $\lim_{t\to \infty }N_{\nu }\left(t\right)=n$, $\lim_{t\to \infty }V_{\nu }\left(t\right)=V$, $T_{\mathrm{cov}}^{CMTS}\leq mT_{S}$. ☐

Let us use a simple example in Figure 1 to illustrate how CMTS works. In Figure 1, nodes A–F are the cluster heads. When the preset sending interval of the sync packet has achieved in cluster heads, the cluster heads will trigger the process of sync message exchange, as in the description of CMTS algorithm. Suppose that node 1 in cluster A owns the biggest hardware clock parameters among all nodes, as shown in Table 2.

According to the update rule in CMTS algorithm, cluster head A would adjust its logic clock parameters to synchronize with node 1 in logic time after two times sync message exchange, because the relative clock skew between two nodes are computing based on at least two sets of adjacent hardware clock. Similarly, after another one sync message exchange between cluster head A and its entire cluster member, node 1, 2, 3, and 4 can synchronize with cluster head A; namely, cluster A needs a total of three times message exchange to realize the time sync. The updated clock parameters are given in Table 3, where the number above the narrow denotes the number of message exchanges. Here, we should notice that it may need two times message broadcasting to achieve sync for the cluster head if it has the maximum clock parameters.

**Figure 2:**
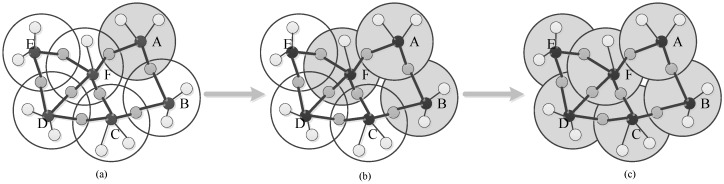
Illustration of inter-cluster time synchronization. (**a**) Time synchronization in cluster A; (**b**) Cluster B and F synchronize with cluster A; (**c**) Cluster C, D, and E synchronize with their adjacent clusters, respectively.

At the same time, other clusters can reach sync with cluster A with the help of overlap nodes, just like the spread of an epidemic, as shown in Figure 2, where a grey circle means the synchronous area. When cluster A has achieved synchronization, it serves as the time source of its adjacent cluster F and B. So, after another at most three times sync message exchange for cluster B and F, respectively, cluster A, B and F can synchronize as shown in Figure 2b, and the sync process for cluster C, D and E is shown in Figure 2c.

### 4.2. Revised-CMTS Method

In CMTS, we suppose that the communication delays are constant. However, many kinds of noises would disturb the link stability, which may severely affect the performance of time synchronization if ignored.Hence, we first analyze the performance of CMTS in the presence of communication delays. Then, the Revised-CMTS is proposed under communication delays.

Consider the communication delays, when node *i* receives a packet from node *j* at time $t_{k}$, it records its clock reading as

$$\tau_{i}\left(t_{k}\right)=\alpha_{i}t_{k}+d_{k}+\beta_{i}$$

Similarly,

$$\tau_{i}\left(t_{k-1}\right)=\alpha_{i}t_{k-1}+d_{k-1}+\beta_{i}$$

where $d_{k}$ is the communication delay. In CMTS, the uplink and downlink between two adjacent nodes is symmetrical—namely, ideal communication link, $d_{k}-d_{k-1}=0$. But in a real network environment, the strict ideal conditions do not exist; namely, $\Delta d_{k}=d_{k}-d_{k-1}\neq 0$. In many early works, $\Delta d_{k}$ is modeled as a special probability distribution, such as an exponential distribution or Gaussian distribution. However, because of the frequent change of the temperature and the humidity of the external environment, as well as the aging hardware, the packet transmission time in Physical Layer (PHY layer) and space link would deviate from the default distribution. So, as a general and practical model, bounded communication delay has been considered in [20]. It assumes that there exists an upper bound *U* on the maximum delay for the reception of a packet; that is,

$$\left|d_{k}-d_{k-1}\right|\leq U,\forall k\geq 1$$

Therefore, the calculation of the relative clock skew in node *i* is

(7) α^ij(tk)=τj(tk)−τj(tk−1)τi(tk)−τi(tk−1)=αj(tk−tk−1)αi(tk−tk−1)+(dk−dk−1)=αijαij(1+ΔdkΔdkΔtkΔtk)(1+ΔdkΔdkΔtkΔtk)

Meanwhile, there exists a lower bound *L* on the minimum interval between the transmissions of two consecutive sync packets; namely,

$$L\leq t_{k}-t_{k-1},\forall k\geq 1$$

From the above equation, we know that α^ij∈αijαij(1+U+ULL)(1+U+ULL),αijαij(1−UULL)(1−UULL). Hence, the previous CMTS method cannot assure the convergence of ${\hat{\alpha }}_{ij}$ and ${\hat{\alpha }}_{ij}$ may approaches infinity when the sync interval approaches the upper bound of communication delays.

At the same time, in CMTS, the update rule of clock skew and offset compensation parameters are based on the relative clock skew. Suppose that the relative clock skew of node *i* and *j* has converged, and *j* has the maximum clock parameters; but with the communication delay, when *i* is synchronizing with *j*, there is

(8) $${\hat{\beta }}_{i}^{\prime }\left(t_{k}^{+}\right)\leftarrow {\hat{\alpha }}_{j}\left(t_{k}\right)\tau_{j}\left(t_{k}\right)+{\hat{\beta }}_{j}\left(t_{k}\right)-{\hat{\alpha }}_{i}\left(t_{k}\right)\tau_{i}\left(t_{k}+d_{k}\right)$$

(9) $${\hat{\beta }}_{i}^{\prime }\left(t_{k-1}^{+}\right)\leftarrow {\hat{\alpha }}_{j}\left(t_{k-1}\right)\tau_{j}\left(t_{k-1}\right)+{\hat{\beta }}_{j}\left(t_{k-1}\right)-{\hat{\alpha }}_{i}\left(t_{k-1}\right)\tau_{i}\left(t_{k-1}+d_{k-1}\right)$$

(10) $$\Delta {\hat{\beta }}^{\prime }={\hat{\beta }}_{i}^{\prime }\left(t_{k-1}^{+}\right)-{\hat{\beta }}_{i}^{\prime }\left(t_{k}^{+}\right)={\hat{\alpha }}_{i}\alpha_{i}\cdot \Delta d_{k}$$

So, even though the logic clock skew is synchronous, the logic clock offset will not synchronize with each other because of the interference caused by communication delays.

Here, by reference to the work in [26], we propose the Revised-CMTS method against the existence of communication delays. It is well known that energy consumption is the major challenge for algorithm design in sensor networks. Additionally, a deployment sink node is a widely used technique in sensor networks used to save energy. So, in Revised-CMTS, the cluster head is used to take on more computing tasks, as well as the sink node. Namely, cluster head is responsible for calculating the relative clock skew and sends the result to cluster members with specific identification. In order to tackle the above problems, we adapt CMTS to Revised-CMTS as in Algorithm 2.

Unlike MTS and CMTS, we estimate the revised relative clock skew from (11) to (12). Note that $\left|d_{k}-d_{k-1}\right|\leq U$, we have $\tau_{i}\left(t_{k}+d_{k}\right)-\tau_{i}\left(t_{k-1}+d_{k-1}\right)-U\leq \alpha_{i}\left(t_{k}-t_{k-1}\right)$, which means that ${\hat{\alpha }}_{ji}^{\prime }\left(t_{k}\right)\leq \alpha_{ji}$. Meanwhile, we set ${\hat{\alpha }}_{ji}\left(1\right)={\hat{\alpha }}_{ji}^{\prime }\left(1\right)$ and update ${\hat{\alpha }}_{ji}\left(t_{k}\right)$ with the maximum value of ${\hat{\alpha }}_{ji}\left(t_{k-1}\right)$ and ${\hat{\alpha }}_{ji}^{\prime }\left(t_{k}\right)$, which indicates that ${\hat{\alpha }}_{ji}\left(t_{k}\right)$ is a non-decreasing function of iteration *k* with an upper bound $\alpha_{ji}$, and will converge after multiple iterations. Similarly, $\tau_{i}\left(t_{k}\right)-U\leq \alpha_{i}t_{k}+\beta_{i}$, then we have $\gamma_{i}\left(t_{k}\right)\geq {\hat{\alpha }}_{j}\left(t_{k}\right)\beta_{j}+{\hat{\beta }}_{j}\left(t_{k}\right)-{\hat{\alpha }}_{i}\left(t_{k}\right)\beta_{i}$, which means that $\gamma_{i}\left(t_{k}\right)$ is a non-increasing function of iteration *k* with a lower bound after the convergence of logic skew. Then, the following update of skew compensation parameter is similar to MTS and CMTS, but the update of offset compensation parameters is in the opposite direction.

Hence, the clock skew and offset compensation under (11)–(18) converges in probability one. The proof of the above update rules is similar to [26], so we will not repeat it here. However. we should point out that the update of logic offset is based on the minimum value, and Revised-CMTS performs the calculation process of revised relative clock skew in cluster heads to save energy consumption of ordinary nodes.

Additionally, for CMTS and Revised-CMTS, each cluster head updates its clock after receiving all of its cluster members’ time information during each iterative process, and each cluster member updates its clock upon receiving the cluster head’s time synchronization. Therefore, the computation complexity of CMTS and Revised-CMTS in cluster head and cluster member are different; namely, $o\left(\left|C_{i}\right|\right)$ for cluster head *i* and $o\left(1\right)$ for cluster member. Meanwhile, clustering techniques have been widely used in WSNs to reduce the communication traffic between the nodes. The computation, storage, communication and power supply ability of cluster-head is usually larger than ordinary nodes. So, it is feasible that cluster-head bears more calculation and communication in sync methods, which has revealed the efficiency on the implementation issue of our method.

**Algorithm 2** Revised-CMTS(1). When cluster head *i* has received two consecutive sync packets from cluster member *j*, it calculates the reciprocal of revised relative clock skew and updates its clock skew and offset compensation parameters as follows:(11)${\hat{\alpha }}_{ji}^{\prime }\left(t_{k}\right)=\frac{1}{{\hat{\alpha }}_{ji}^{\prime }\left(t_{k}\right)}=\frac{\tau_{i}\left(t_{k}+d_{k}\right)-\tau_{i}\left(t_{k-1}+d_{k-1}\right)-U}{\tau_{j}\left(t_{k}\right)-\tau_{j}\left(t_{k-1}\right)}.$
(12)$${\hat{\alpha }}_{ji}\left(t_{k}\right)\leftarrow \max\left\{{\hat{\alpha }}_{ji}\left(t_{k-1}\right),{\hat{\alpha }}_{ji}^{\prime }\left(t_{k}\right)\right\}.$$
(13)α^i(tk+)←maxα^i(tk−1),α^j(tk)α^j(tk)α^ji(tk)α^ji(tk).
(14)$$\gamma_{i}\left(t_{k}^{+}\right)\leftarrow \min\left\{\gamma_{i}\left(t_{k}\right),{\hat{\alpha }}_{j}\left(t_{k}\right)\tau_{j}\left(t_{k}\right)+{\hat{\beta }}_{j}\left(t_{k}\right)-{\hat{\alpha }}_{i}\left(t_{k}\right)\left(\tau_{i}\left(t_{k}\right)-U\right)\right\}.$$
(15)${\hat{\beta }}_{i}\left(t_{k}^{+}\right)\leftarrow \min\{{\hat{\beta }}_{i}\left(t_{k}\right),\gamma_{i}\left(t_{k}^{+}\right)\}.$ (2). When cluster member *j* receives sync packets from cluster head *i*, it updates its clock skew and offset compensation parameters as follows:(16)${\hat{\alpha }}_{j}\left(t_{k}^{+}\right)\leftarrow \max\left\{{\hat{\alpha }}_{j}\left(t_{k-1}\right),{\hat{\alpha }}_{i}\left(t_{k}\right){\hat{\alpha }}_{ji}\left(t_{k}\right)\right\}.$
(17)$$\gamma_{j}\left(t_{k}^{+}\right)\leftarrow \min\left\{\gamma_{j}\left(t_{k}\right),{\hat{\alpha }}_{i}\left(t_{k}\right)\tau_{i}\left(t_{k}\right)+{\hat{\beta }}_{i}\left(t_{k}\right)-{\hat{\alpha }}_{j}\left(t_{k}\right)\left(\tau_{j}\left(t_{k}\right)-U\right)\right\}.$$
(18)$${\hat{\beta }}_{j}\left(t_{k}^{+}\right)\leftarrow \min\{{\hat{\beta }}_{j}\left(t_{k}\right),\gamma_{j}\left(t_{k}^{+}\right)\}.$$

## 5. Performance Analysis and Simulation Results

Based on the convergence speed, scalability and communication overhead, we perform analysis and simulations on current distributed algorithms ATS [21] and MTS [12], and cluster-based algorithm CCTS [18], with comparison to CMTS in convergence rate and communicate overhead. Then, the simulation results of CMTS and Revised-CMTS under bounded communication delays are given out.

### 5.1. Performance Analysis

#### 5.1.1. Analysis of CMTS

ATS and CCTS use the average consensus to achieve synchronization, but cannot realize synchronous convergence of skew and offset. While, MTS and CMTS are based on maximum consensus, they can update the skew and offset at the same time. So, the convergence rate of average-based methods is slow. By contrast, in CCTS and CMTS—which have incorporated cluster technique—a cluster head can obtain all of the member’s time information by one message exchange, which can improve the convergence speed effectively.

Due to the restriction of hardware resources and battery-power, low energy consumption is important to prolong the life of IWSNs. Therefore, the research of time synchronization algorithms must take energy cost into account. Meanwhile, communication energy consumption occupies main energy cost in time synchronization. By referencing [12], we analyze the communication energy consumption by the number of broadcast.

With reference to [12], the convergence speed and broadcast times of MTS is given by

(19) $$T_{COV}^{MTS}\leq B\left(n-1\right)$$

(20) $$N_{c}^{MTS}\leq \frac{B(n-1)}{T}\sum_{i\in (1,n)}\alpha_{i}$$

where *B* is a time interval that guarantees the connecting of the networks, indicating that there exists one message exchange during the period, and $N_{c}^{MTS}$ is the broadcast numbers of all nodes.

Based on CMTS algorithm, the broadcast times of CMTS are

(21) $$N_{c}^{CMTS}\leq \frac{m\cdot T_{S}}{T}\sum_{i\in (1,m)}|C_{i}|\alpha_{i}$$

For (6), (19)–(21), we can assume that $3B\geq T_{S}$, which is reasonable because $T_{S}$ is a time interval that guarantees at most three times message exchange in the networks. In a given IWSNs, $m<\frac{n-1}{3}$, which is reasonable because the number of nodes in a cluster is usually greater than 3; hence, the upper bond of convergence time is lower in CMTS. Meanwhile, $n=|\cup_{i\in (1,m)}C_{i}|$; hence, the upper bond of broadcast times is also lower in CMTS when $\alpha_{i}$ is fixed.

#### 5.1.2. Analysis of Revised-CMTS

In Revised-CMTS, in order to reduce the influence of communication delays, we construct two non-increasing and non-decreasing sequence by making a change to CMTS. Additionally, the revised relative clock skew ${\hat{\alpha }}_{ji}\left(t_{k}\right)$ converges at $d_{k}-d_{k-1}-U=0$—namely, $d_{k}-d_{k-1}=U$—which indicates that $d_{k}=U_{upper}$ and $d_{k-1}=U_{lower}$. Hence, ${\hat{\alpha }}_{ji}\left(t_{k}\right)=\alpha_{ji}$ and $\gamma_{i}\left(t_{k}\right)={\hat{\alpha }}_{j}\left(t_{k}\right)\beta_{j}+{\hat{\beta }}_{j}\left(t_{k}\right)-{\hat{\alpha }}_{i}\left(t_{k}\right)\beta_{i}$, which means that the logic offset and logic skew converge at the same time. Accordingly, the convergence time of the algorithm depends on the probability that the lower bound and upper bound of communication delays appear successively; namely, a higher probability generates a faster convergence speed.

From the above analysis, one can infer that the convergence of Revised-CMTS heavily relies on ${\hat{\alpha }}_{ji}\left(t_{k}\right)$. However, it is generally known that the transmission of messages between nodes is independent of each other, so the calculation of ${\hat{\alpha }}_{ji}\left(t_{k}\right)$ in both sides would make a smaller convergence probability. Meanwhile, it does not need repeated calculation of relative clock skew because αji=11αijαij, which can be performed in cluster heads due to their powerful calculation and storage. Therefore, the method is implemented in Revised-CMTS to further improve the convergence speed.

### 5.2. Simulation Results

We perform simulation and analysis on CMTS and Revised-CMTS methods in Matlab R2015a. Assume that the clock skew and offset are randomly selected from the set (0.8, 1.2) and (0, 0.4), respectively, and the broadcast period is 1 s [12]. Set the skew and offset compensation parameters to 1 and 0, respectively. Because most of the current low-power wireless sensor nodes provide a 32,768 Hz crystal oscillator, we set 1 tick = 30.5 μs. To ensure that the cluster head located in the center of the 1 × 1 grid can communication with all of its cluster members, the maximum communication range of each node is $\sqrt{0.5}$. In the following results, we will compare our CMTS method with ATS, MTS, and CCTS algorithm, and analyze the convergence of Revised-CMTS under bounded communication delays.

Throughout the simulation, for cluster-based methods (including CCTS and CMTS), nodes are randomly distributed in the network, which means that the cluster heads are located in the center of each 1 × 1 grid, and the others are deployed randomly. This is feasible that the cluster heads are usually pre-placed in IWSNs.

#### 5.2.1. Without Communication Delays

**Figure 3:**
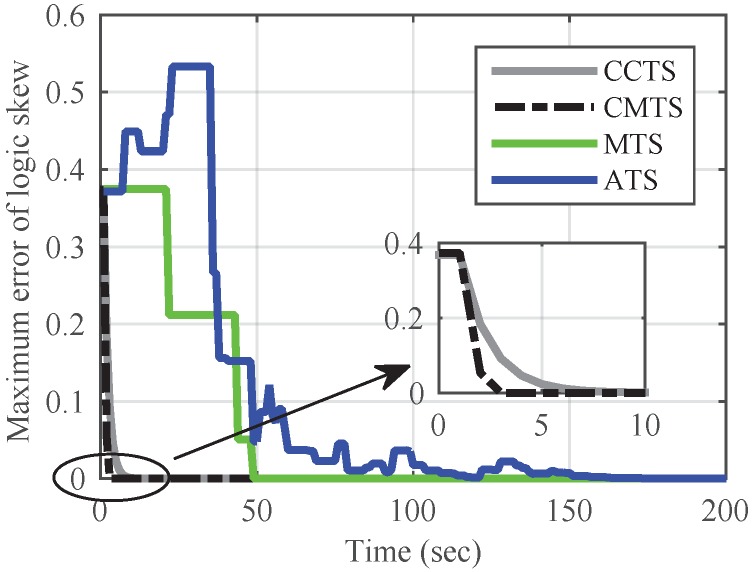
Comparison of convergence speed of skew in intra-cluster time synchronization among the proposed Cluster-based Maximum consensus Time Synchronization (CMTS) and the current methods.

Put 20 nodes in a 1 × 1 grid IWSNs randomly. Figure 3 shows the convergence speed of skew synchronization in intra-cluster synchronization. As expected, the convergence speed of CMTS and ATS are the fastest and lowest, respectively. For CCTS, the convergence time of CCTS’s intra-cluster synchronization satisfies $T_{e}\left(\frac{\left|C_{i}\right|+1}{\left|C_{i}\right|}+1\right)\leq T_{ccts}\leq T_{e}\left(8{\left(\frac{\left|C_{i}\right|+1}{\left|C_{i}\right|}\right)}^{2}+1\right)$, where $T_{e}$ is the time interval that guarantees one message exchange in the cluster [18]. Hence, $3T_{e}\leq T_{ccts}\leq 9T_{e}$. We assume that $B=T_{e}$, as *B* also indicates that there exists one message exchange during the period. Since there are 20 nodes here, the upper bound of $T_{COV}^{MTS}$ is $19T_{e}$ based on the Equation (19). Therefore, the clustered CMTS and CCTS reveal a better convergence property than ordinary distributed methods, with the help of cluster head to collect the network time information.

**Figure 4:**
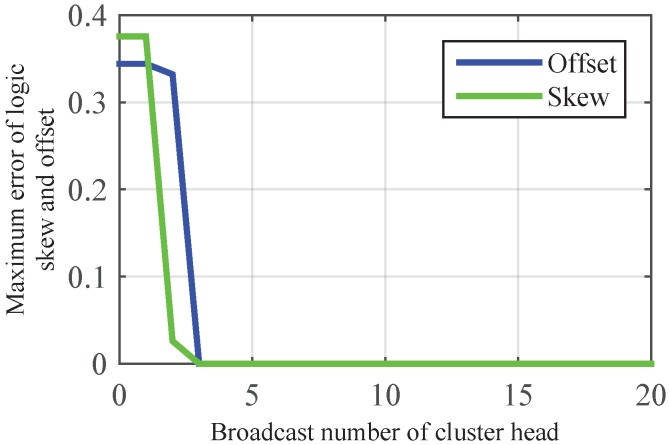
Illustration of convergence speed of logic clock skew and offset by using CMTS in intra-cluster time synchronization.

**Figure 5:**
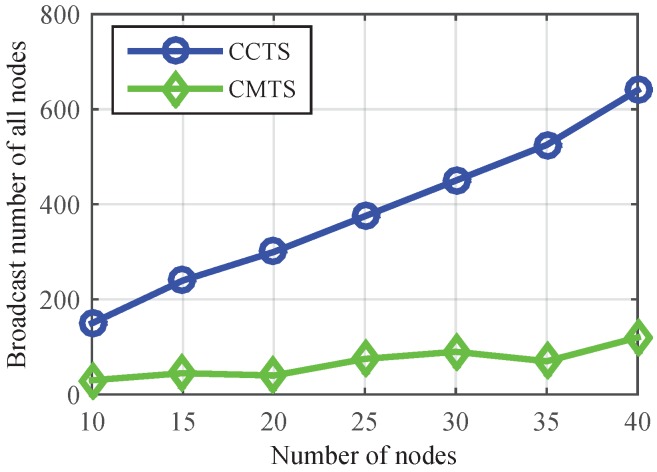
Comparison of Clustered Consensus Time Synchronization (CCTS) and CMTS on communication overhead in intra-cluster time synchronization.

As shown in Figure 4, CMTS could achieve skew and offset synchronization simultaneously after three times broadcast of cluster head, as the cluster head can obtain the maximum value of skew via two times message exchange, and transmits the information to its members on the third time. With the increasing of nodes in a single cluster, Figure 5 shows that the number of broadcasts in CMTS is much less than CCTS, which proves the lower communication overhead of CMTS. This is because—in CCTS, the offset compensation is started after the implementation of the skew compensation, and the acquisition of average value needs multiple iterations.

**Figure 6:**
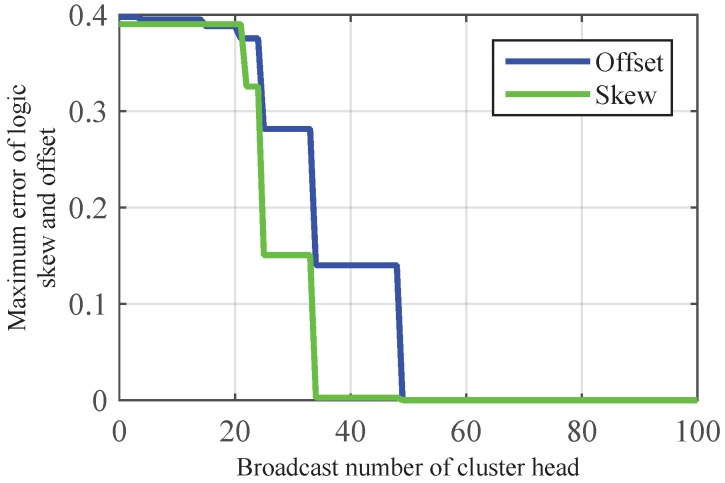
Comparison between skew and offset by using CMTS in inter-cluster time synchronization.

Consider a network with 100 nodes. Assume that the network is randomly divided into 9 clusters in a 3 × 3 grid area. From Figure 6, it can be seen that the skew and offset synchronization can be achieved at the same time, which indicates that CMTS has inherited MTS’s superiority.

**Figure 7:**
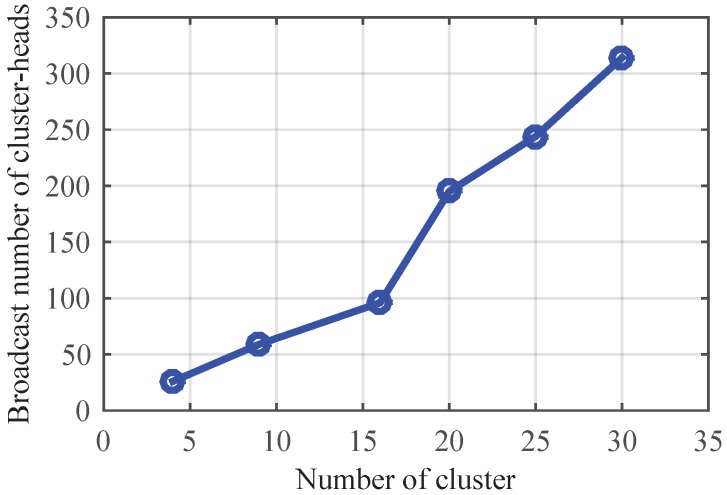
Communication overhead of CMTS in inter-cluster time synchronization.

Figure 7 shows the effect of the number of clusters on algorithm performance. We put 50, 100, 200, 300, 400 and 500 nodes in several networks, and divide the networks into 4, 9, 16, 20, 25 and 30 clusters, respectively. From Figure 7, we can see that the broadcast times of cluster heads are almost a linear function with the increasing of clusters in a network, which proves CMTS’s scalability. Simultaneously, it can be seen that fewer clusters lead to less communication overhead. Therefore, clustering is an effective way to reduce the energy cost of sync method.

Based on the above analysis and simulations, we can see that this paper has presented a CMTS method with superior performance of communication overhead and convergence rate over existing synchronization methods.

#### 5.2.2. With Communication Delays

Taking the bounded communication delays into consideration, we compare the convergence property of logic clock skew and offset in CMTS and Revised-CMTS, and the network environment is the same as the above section. The communication delay is randomly selected from 0–0.02, and set the upper bound and lower bound achieved at the 226th and 227th broadcast in cluster head, respectively.

**Figure 8:**
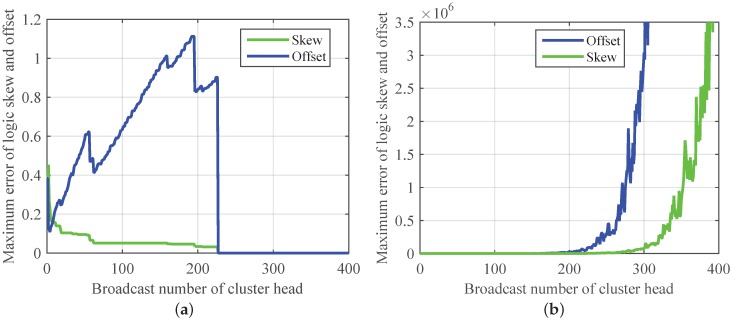
Comparison the property of Revised-CMTS and CMTS in intra-cluster time synchronization. (**a**) Revised-CMTS; (**b**) CMTS.

First, we realize Revised-CMTS and CMTS in intra-cluster time synchronization. As shown in Figure 8, Revised-CMTS has a faster convergence speed and higher synchronization accuracy than those of CMTS, and CMTS cannot achieve synchronization. From the Figure 8a, we can see that the error of logic skew drops gradually and converges to zero at the 227th broadcast. For logic offset, the error fluctuates and presents an increasing trend at first, but eventually achieves convergence. The results validate the effectiveness of Revised-CMTS. Here, we should notice that the sooner the lower and upper bound delays successively appear, the faster the Revised-CMTS converges.

Finally, we analyze the performance of Revised-CMTS in inter-cluster sync. Put 50 nodes into a 2 × 2 network with four clusters randomly. Here, the upper bound and lower bound of the communication delays achieve at the (126·*k*)th and (126·*k*–1)th broadcast in cluster heads, respectively.

**Figure 9:**
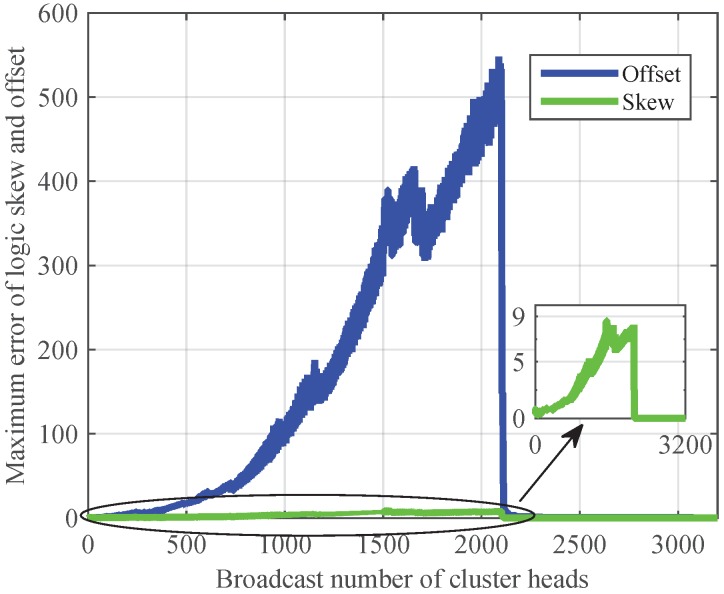
Comparison the property of Revised-CMTS and CMTS in inter-cluster time synchronization.

The result is given in Figure 9. From the previous simulation parameters setting and Figure 8a, we can see that each cluster realizes synchronization around the (126·*k*)th broadcast. So, for IWSNs with four clusters, Revised-CMTS would converge after the 504th (126 × 4) broadcast of a cluster head, that is, more than 2016 (504 × 4) times broadcast for all cluster heads, which is shown in Figure 9. At the same time, time synchronization for cluster-based IWSNs is achieved cluster by cluster, so the errors of both the node’s logic clock skew and offset fluctuates and presents an increasing trend at first. It also reveals that the number of clusters has a negative impact on Revised-CMTS.

## 6. Conclusions

In this paper, we propose a novel time synchronization method called CMTS for IWSNs, based on the maximum consistency theory and incorporating the clustering technique. This method is superior to existing works in convergence rate and communication overhead. It can achieve linear convergence in finite time, with scalability against clusters’ number. At the same time, a Revised-CMTS has been given out under bounded communication delays. Simulation results and theoretical analysis show that the number of clusters has a great impact on the convergence time and communication overhead. That is, the less clusters, the faster convergence speed and the less energy consumption.

However, the communication range and the location of nodes will change over time because of the energy reduction and movement, which will lead to the change of clusters, as well as node failure. Therefore, extensive work is still necessary to deal with the dynamic topology structure. On the other hand, the communication delays would vary with the change of external environment; hence, the effectiveness of the Revised-CMTS would reduce because of the preset upper bound of the communication delays. It is necessary to design a time synchronization method against dynamic communication delays.

## Figures and Tables

**Table 1:** Notation definitions.

Symbol	Definitions
$\tau_{i}\left(t\right)$	the local clock reading of node *i* at time *t*;
$\alpha_{i}$	the local clock skew of node *i*;
$\beta_{i}$	the local clock offset of node *i*;
${\hat{\tau }}_{i}\left(t\right)$	the logical clock reading of node *i* at time *t*;
${\hat{\alpha }}_{i}\left(t\right)$	the skew compensation parameter of node *i* at time *t*;
${\hat{\beta }}_{i}\left(t\right)$	the offset compensation parameter of node *i* at time *t*;
*n*	the number of nodes;
*m*	the number of clusters;
$\left|C_{i}\right|$	the number of nodes in cluster *i*;
$t^{+}$, $t_{k}^{+}$	the time just after updating at time *t*, $t_{k}$;
$\alpha_{ij}$	the relative clock skew between node *i* and *j*;
*U*	the upper bound on the communication delay;
*L*	the lower bound on the interval between the transmissions of two sync packets.

**Table 2:** The initial clock parameters.

Node	$\alpha_{i}$	$\beta_{i}$	${\hat{\alpha }}_{i}$	${\hat{\beta }}_{i}$	${\hat{\alpha }}_{i}\alpha_{i}$	${\hat{\alpha }}_{i}\beta_{i}+{\hat{\beta }}_{i}$
A	0.4	0.7	1	0	0.4	0.7
**1**	**0.8**	**0.9**	**1**	**0**	**0.8**	**0.9**
2	0.5	0.3	1	0	0.5	0.3
3	0.6	0.7	1	0	0.6	0.7
4	0.3	0.5	1	0	0.3	0.5

**Table 3:** Clock Parameters Update after Sync.

Node	$\alpha_{i}$	$\beta_{i}$	${\hat{\alpha }}_{i}$	${\hat{\beta }}_{i}$	${\hat{\alpha }}_{i}\alpha_{i}$	${\hat{\alpha }}_{i}\beta_{i}+{\hat{\beta }}_{i}$
A	0.4	0.7	$1\overset{2}{\to }2$	$0\overset{2}{\to }0.5$	$0.4\overset{2}{\to }0.8$	$0.7\overset{2}{\to }0.9$
1	0.8	0.9	1	0	0.8	0.9
2	0.5	0.3	$1\overset{3}{\to }1.6$	$0\overset{3}{\to }0.48$	$0.5\overset{3}{\to }0.8$	$0.3\overset{3}{\to }0.9$
3	0.6	0.7	$1\overset{3}{\to }1.33$	$0\overset{3}{\to }0.03$	$0.6\overset{3}{\to }0.8$	$0.7\overset{3}{\to }0.9$
4	0.3	0.5	$1\overset{3}{\to }2.67$	$0\overset{3}{\to }0.435$	$0.3\overset{3}{\to }0.8$	$0.5\overset{3}{\to }0.9$
